# Building a virtual ligand screening pipeline using free software: a survey

**DOI:** 10.1093/bib/bbv037

**Published:** 2015-06-20

**Authors:** Enrico Glaab

**Keywords:** virtual screening, docking, protein–ligand binding, ADMETox, off-target effects, workflow management

## Abstract

Virtual screening, the search for bioactive compounds via computational methods, provides a wide range of opportunities to speed up drug development and reduce the associated risks and costs. While virtual screening is already a standard practice in pharmaceutical companies, its applications in preclinical academic research still remain under-exploited, in spite of an increasing availability of dedicated free databases and software tools. In this survey, an overview of recent developments in this field is presented, focusing on free software and data repositories for screening as alternatives to their commercial counterparts, and outlining how available resources can be interlinked into a comprehensive virtual screening pipeline using typical academic computing facilities. Finally, to facilitate the set-up of corresponding pipelines, a downloadable software system is provided, using platform virtualization to integrate pre-installed screening tools and scripts for reproducible application across different operating systems.

## Introduction

In the pharmaceutical industry, computational techniques to screen for bioactive molecules have become an established complement to classical experimental high-throughput screening methods. Previous success stories have shown that using virtual screening approaches can help to reduce the required time and costs for drug development projects and mitigate the risk for late-stage failures (e.g. *in silico* techniques were instrumental in the development of the HIV integrase inhibitor Raltegravir [1], the anticoagulant Tirofiban [2] and the influenza drug compound Zanamivir [3]). In recent years, the combination of increasing computing power, improved algorithms and a wider availability of relevant software tools and data repositories has made preclinical drug research using virtual screening more feasible for academic laboratories. However, setting up an efficient and effective screening pipeline is still a major challenge, and a greater awareness about freely available screening, quality control and workflow management software published in recent years would help to more fully exploit the potential of *in silico* screening.

This review discusses the recent progress in screening based on receptors and ligands, with a focus on free software tools and databases as alternatives to commercial resources. New developments in the field (e.g. covalent docking, novel machine learning approaches for binding affinity prediction and automated workflow management software) are covered in combination with practical advice on how to build a typical screening pipeline and control quality and reproducibility. As a generic guideline for screening projects with an already chosen protein drug target of interest (see [4] for an overview of target identification approaches not covered here), a comprehensive framework and pipeline for virtual small-molecule screening is described, providing examples of free software tools for each step in the process. To facilitate the set-up of a corresponding screening pipeline and integrate pre-installed public tools within a unified software framework, a downloadable cross-platform software for reproducible virtual screening using the Docker system is provided (see section on ‘Generic screening framework and workflow management’ below and the website https://registry.hub.docker.com/u/vscreening/screening).

## Data collection/molecular structure and interaction databases

### Protein structure databases

The availability of 3D structure data for a target protein of interest is a major benefit for virtual screening studies, although purely ligand-based screening methods may provide an alternative if no suitable target structure can be obtained (see section on ligand-based screening below). An overview of the main public repositories for experimentally derived and *in silico* modelled protein structures is given in Table 1. Among these, the Protein Data Bank (PDB) [5] is the standard international archive for experimental structural data of biological macromolecules, covering ∼107 000 structures as of March 2015. It provides access to the most comprehensive collection of public X-ray crystal structures and is the default resource to obtain protein structures for receptor-based screening. In spite of the rapid growth of the PDB, almost doubling in size over the past six years, many protein families are still not covered by a representative structure, and even in an ideal model scenario, the coverage is not expected to reach 80% before 2020 and 90% before 2027 [6]. As the structures in the PDB are biased towards proteins that can be purified and studied using X-ray crystallography, nuclear magnetic resonance (NMR) spectroscopy and electron microscopy, certain types of proteins, including pharmacologically important membrane proteins, are underrepresented in the database. Importantly, the quality of PDB structures is also restricted by limitations of the experimental methodologies, e.g. hydrogen atoms and flexible components cannot be resolved via X-ray diffraction, and NMR techniques usually provide lower resolutions than X-ray crystallography. Often the experimental methods fail to determine the entire protein structure, and many PDB files have missing residues or atoms (see section on protein structure pre-processing and quality control for guidelines on how to deal with these and other potential shortcomings of PDB files).

**Table 1.** The main public repositories for experimentally derived and *in silico* modelled protein structures, including details on content type, approximate number of current entries and accessibility

Database	Content type	Approx. no. of entries	Webpage
PDB [5]	X-ray, Solution NMR, Electron Microscopy, among others	107k (∼95k X-ray, ∼11k Solution NMR, ∼700 Electron Microscopy, ∼300 others)	http://www.rcsb.org
ModBase [8]	3D protein models from comparative modelling	3.8 million models	http://salilab.org/modweb
SWISS-MODEL Repository [9]	3D protein models from homology modelling	3.2 million models	http://swissmodel.expasy.org/repository/
Protein Model Portal (PMP) [10]	Integration of modelled structures from multiple servers	21.8 million models	http://www.proteinmodelportal.org
Structural Biology Knowledgebase (SBKB) [13]	PDB structures and associated homology models	See PDB and PMP database	http://sbkb.org

If no suitable experimental structure for molecular docking simulations can be identified for a chosen target protein, a binding site structural model may alternatively be derived from comparative modelling, if a template protein with close homology to the target is available. While the performance of docking simulations using homology models will depend on the sequence similarity of the template(s) to the target protein, the quality of the template structure(s) and the modelling approach, the analyses from a previous large-scale validation study by Oshiro *et al*. can provide a guideline on the results to be expected in different scenarios [7]. The authors assessed the performance of docking into homology models using CDK2 and factor VIIa screening data sets, and found that when the sequence identity between the model and template near the binding site is greater than ∼50%, roughly 5 times more active compounds are identified than by random chance (a performance that was comparable with docking into crystal structures according to their observations). Their publication provides a plot of the enrichment of true-positive discoveries versus the percentage sequence identity between the template and target, which can serve as an orientation for future studies. Large-scale collections of existing protein structure models, including ModBase [8], SWISS-MODEL [9] and PMP [10], are listed in Table 1 as resources for proteins not covered by known experimental structures. Alternatively, new comparative models for specific target proteins can be generated using dedicated homology modelling tools, reviewed in detail elsewhere [11]. To prevent spurious results due to low-quality models, users can estimate the accuracy of docking simulations based on homology models a priori via established indices for model quality assessment [12].

### Small-molecule databases

Screening projects to identify new selective and potent inhibitors of a chosen target protein typically use large-scale compound libraries containing several thousands or millions of small molecules to start the filtering process. Depending on the goal and type of the study (e.g. drug development, toxin identification, pesticide development), the compound library may contain already known drug substances for repositioning, synthetic substances similar to lead or drug compounds for subsequent structural optimization or other natural or xenobiotic compounds. To design suitable compound libraries in terms of the type, number and commercial availability of the included molecules, access to large, structured and well-annotated repositories of small molecules is needed. Some of the most comprehensive free databases include ZINC [14] (35 million compounds), PubChem [15] (64 million compounds) and ChemSpider [16]. While many of the largest databases (e.g. ChemNavigator [17] with 60 million compounds) are commercial and only provide restricted data access for academic research, in recent years, public initiatives and vendors of small-molecule compounds have made several structured libraries publicly accessible. When downloading structure files from these repositories, users should note that they are usually not designed for virtual screening purposes and multiple pre-processing and format conversions are required. An exception is the ZINC database, a dedicated data repository for virtual screening [14], providing unrestricted access to already pre-processed and filtered structures. However, even when using a collection of already pre-processed ligands, it is often recommendable to test alternative pre-processing methods depending on the following analysis pipeline (see section on ligand pre-processing below).

### Protein–ligand interaction and binding affinity databases

For most proteins, only few or no small-molecule binders with high affinity (in the nanomolar or low micromolar range) and selectivity are already known from previous studies. Moreover, the reported affinities often vary significantly depending on the used measurement technique [18]. Proteins with multiple known and well-characterized binders for the same binding pocket, however, cover several targets of biomedical interest, and the existing data can provide opportunities for identifying new structurally similar molecules with improved selectivity and affinity via ligand-based screening (see dedicated section below). Moreover, existing interaction and binding affinity data are a useful resource for identifying or predicting off-target effects [19]. To collect information on the known protein–ligand interactions for a receptor or small molecule of interest, Table 2 lists the main relevant databases, most of which are publicly accessible. Drug2Gene [29], the currently most comprehensive meta-database, may provide a first point of reference for most types of queries. Other repositories have a more specific scope, e.g. PDBbind [30] focuses exclusively on binding affinity data from protein–ligand complexes in the PDB. As the databases in Table 2 are updated at different intervals and contain many non-overlapping entries, a study requiring a comprehensive coverage of known interactions for a target molecule should collect current data from all accessible repositories. Importantly, issues in data heterogeneity, redundancies and biases in the database curation process can result in biased *in silico* models of drug effects, and strategies proposed to address or alleviate these problems include the use of model-based integration approaches (e.g. KIBA [31]) and sophisticated data curation and filtering processes (e.g. the procedure proposed by Kramer *et al.* [32], which includes the calculation of several objective quality measures from differences between reported measurements).

**Table 2.** Overview of protein–ligand interaction and binding affinity databases with details on the approximate current number of entries and public accessibility

Database	Approx. no. of entries	Free for academia	Webpage
Drug2Gene [29]	4.4 million	yes	http://www.drug2gene.com
BindingDB [18]	1.1 million	yes	http://www.bindingdb.org
SuperTarget [24]	330k	yes	http://insilico.charite.de/supertarget
PDSP Ki Database [25]	55k	yes	http://pdsp.med.unc.edu/kidb.php
Binding MOAD [26]	23k	yes	http://www.bindingmoad.org
PDBbind [30]	11k	yes	http://www.pdbbind.org.cn
Thomson Reuters MetaDrug	700k	no	http://thomsonreuters.com/metadrug/

## Data pre-processing/filtering and quality control

Quality checking and pre-processing of molecular structure files is a critical step in virtual screening projects, typically involving a combination of manual data inspection and automated processing via programming scripts. In the following sections, an overview is provided of the main steps and software tools for quality control and pre-processing of protein receptor and small-molecule structures and filtering of the compound library.

### Protein structure pre-processing and quality control

A typical procedure for the preparation of protein structures for virtual screening consists of the following steps: (1) select the protein and chain for docking simulations and determine the relevant binding pocket; (2) quality control (check for format errors, missing atoms or residues and steric clashes); (3) determine missing connectivity information, bond orders and partial charges/protonation states (preferably, multiple possible states should be considered during docking simulations); (4) add hydrogen atoms; (5) optimize hydrogen bonds; (6) create disulphide bonds and bonds to metals (adjust partial charges, if needed); (7) select water molecules to be removed (preferably, multiple selections should be considered during docking simulations); (8) fix misoriented groups (e.g. amide groups of asparagine and glutamine, the imidazole ring in histidines; adjust partial charges, if needed); (9) apply a restrained protein energy minimization (run a minimization while restraining heavy atoms not to deviate significantly from the input structure; receptor flexibility should still be taken into consideration during the docking stage) and; (10) final quality check (repeat the quality control for the pre-processed structure). Sastry *et al*. performed a comparative evaluation of different pre-processing steps and parameters, suggesting that each of the common optimization steps is relevant in practice and that, in particular, the H-bond optimization and protein minimization procedures, which are sometimes left out in automated pre-processing tools, can improve the final enrichment statistics [33]. Interestingly, their results also indicate that retaining water molecules for protein preparation and then eliminating them before docking was inconsequential as compared with removing water molecules prior to any preparation steps (however, they did not consider alternative selections of water molecules during the docking stage, see discussion below). While Sastry *et al*. focus on commercial pre-processing software for the docking tool GLIDE [34], in the following paragraph, alternative methods and tools for the different pre-processing steps are discussed.

At first, the user chooses the protein structure and chain for docking (or ideally, multiple available structures for the target protein are used to run docking simulations in parallel) and determines the relevant binding site. Should the binding site not be known from previous crystallized protein–ligand complexes, several binding pocket prediction methods are available, e.g. MetaPocket [35], DoGSiteScorer [21], CASTp [36] and SplitPocket [20] (see [28] for a review of related approaches). Next, a quality control is necessary, as protein crystal structures in public repositories like the PDB often contain errors or missing residues (see the section on protein structure databases). Only some of the issues can be addressed by automated pre-processing tools, and protein structure files should therefore first be checked manually. PDB files can be opened in a simple text editor and often contain important remarks on shortcomings of the corresponding structure, e.g. a list of missing residues. Missing or mislabelled atoms (not conforming to the IUPAC naming conventions [22]) in residues, unusual bond lengths and steric clashes can be identified via dedicated quality checking tools, e.g. PROCHECK [23], WHAT_IF [27], Verify3D [37] and PDB-REDO [38]. Moreover, by visualizing the combinations of backbone dihedral angles ψ and φ of residues in a 2D graph, known as the Ramachandran plot, users can identify unrealistic conformations in comparison with typically observed ranges of ψ–φ combinations [39]. Additional manual inspection of a protein structure in a molecular file viewer, e.g. UCSF Chimera [40], PyMOL [41], VMD [42], Yasara [43], Rasmol [44], Swiss PDB Viewer [45] and BALLView [46], should be conducted as well, because, in particular, older PDB files often do not conform to the standard format, resulting in unpredictable errors in downstream analyses. Molecular visualization tools like BALLView also allow the user to add missing hydrogens and optimize their positions, remove ligands from complex structures and apply an energy minimization (however, instead of using a static minimized structure, the user should preferably apply docking approaches that account for receptor flexibility; see section on screening using receptor structures below). Selecting the water molecules to be removed is more difficult, as some of them could contribute significantly to protein–ligand interactions, and this may depend on the specific ligand. Although this task still remains a challenge, dedicated approaches are available, e.g. as part of the Relibase+ software, the WaterMap (http://www.schrodinger.com/WaterMap.php) and AcquaAlta [47] method. Preferably, different combinatorial possibilities to include or exclude water molecules should be explored during the docking procedure, in spite of increased runtimes. Similar considerations apply to the protonation states of residues in the active site, which may vary depending on the ligand and should ideally be chosen separately for each docking pose (e.g. using the Protonate 3D software [48] or the scoring function in the eHITS docking software [49]). Moreover, flipped side-chain conformations for His, Gln and Asn residues may need to be adjusted to improve the interactions with neighbouring groups (e.g. using the H++ software [50]). After a final energy minimization, the resulting structure should be checked again using quality control tools (see above).

If multiple crystal structures are available for the target protein, users are advised to select the input for docking simulations not only by comparing structures in terms of resolution, but also domain and side chain completeness, presence of mutations and errors annotated in the structure file (ideally, docking runs will be performed with multiple available structures to compare the results). If on the contrary, no experimental or previously modelled structure of sufficient quality is available for the target protein, potential alternatives may be to use ligand-based screening (see dedicated section below) or to create a new homology model (see [51] for a review of corresponding software). Even when using *in silico* modelled structures, the pre-processing and quality control tools mentioned above should still be applied to check the suitability of the input for the following analyses.

### Ligand pre-processing and pre-filtering of the compound library

Pre-processing of structure files is not only essential for macromolecular target proteins but also for small-molecule compounds. Large-scale compound collections are often stored in compact 1D- (e.g. SMILES) or 2D-formats (e.g. SDF), so that 3D co-ordinates first have to be generated and hydrogen atoms added to the structure. Apart from format conversion tools, such as OpenBabel [52], dedicated ligand pre-processing methods are available to generate customized compound libraries, including tautomeric, ionization and stereochemical variants, and optionally to perform energy minimization (e.g. the software packages LigPrep [53], Epik [54] and SPORES [55]). Specific protonation states and partial charges are typically assigned during the docking stage because they should be consistent for the protein and ligand (a wide range of methods for protonation and partial charge assignment are available and have previously been compared in terms of their benefits for binding affinity estimation [56]).

To avoid prohibitive runtimes for a docking screen against all compounds in a public database, the initial compound collection is typically pre-filtered in accordance with the goals and constraints of the study. For example, compounds that are too large to fit into the targeted binding pocket should be filtered out immediately. Moreover, compounds can be pre-filtered in terms of their ‘drug-likeness’ properties, e.g. using ‘Lipinski’s rule of five’, or related rule sets [57, 58], or in terms of their structural and chemical similarity to already known binding molecules for the target (see section on ligand-based screening). Ligand similarity calculations may also help to remove highly similar structures from a library, making it more compact while retaining a wide coverage of diverse molecules. Relevant tools for compound library design include Tripos Diverse Solution, Accelrys Discovery Studio, Medchem Studio, ilib diverse and the open-source software ChemT [59]. Finally, fast methods to predict bioavailability and toxicity properties of small molecules (see corresponding section on ADMETox filtering below) may also be applied at this stage to filter out compounds with unwanted properties early in the screening process.

## Compound screening and analysis

### Receptor-based screening

If an experimentally derived structure or a high-quality homology model is available for a target protein of interest, receptor-based screening approaches can be applied to predict and rank small molecules from a compound library as putative binders in the protein’s active site. For this purpose, fast molecular docking simulations are used to model and evaluate possible binding poses for each compound. After the binding pocket has been defined and the structure has been pre-processed (see section on protein structure pre-processing), typical docking programs exploit three types of techniques to evaluate large numbers of compounds efficiently:

- compact structure representations (to reduce the size of the search space);
- efficient search space exploration methods (to identify possible docking poses); and
- fast scoring functions (to rank compounds in terms of estimated relative differences in binding affinity).

Dedicated structure representations for molecular docking usually restrict the search space to the receptor binding pocket (as opposed to ‘blind docking’, used when the location of the binding site is unknown) and replace full-atom models by more simplified representations. These include geometric surface representations like spheres [60, 61], Voronoi tessellation or triangulation-based representations (e.g. in BetaDock [62]), grid representations in which interaction potentials of probe atoms are mapped to points on a grid with adjustable coarseness (e.g. in the AutoDock software [63, 64]) or a reduction to points and vectors reflecting critical properties for the interaction with the ligand (e.g. the LUDI representation [65] used in FlexX [66]). Apart from the structure representation, the size of the search space also depends on the extent to which structural flexibility of the ligand and receptor is taken into account. While the consideration of ligand flexibility has become a standard in molecular docking since the introduction of the FlexX software [66], accounting for receptor flexibility and conformational adjustments in the binding pocket upon ligand binding is still a major challenge due to the significant increase in degrees of freedom to be explored. However, depending on the targeted protein family, protein flexibility can often have a decisive influence on binding events and is a major limiting factor for successful screening. Two main generic models have been proposed to describe protein conformational changes upon binding events: the ‘induced-fit’ model, in which the interaction between a protein and its binding partner induces a conformational change in the protein, and the ‘conformational selection’ model (also referred to as population selection, fluctuation fit or selected fit model), in which, among the different conformations assumed by the dynamically fluctuating protein, the ligand selects the most compatible one for binding [67, 68]. Current computational techniques to address receptor flexibility include the use of multiple static receptor representations that reflect different conformations (a strategy known as ‘ensemble docking’) [69], the search for alternative amino acid side-chain conformations at the binding site using rotamer libraries [70, 71] and the representation of flexibility via relevant normal modes [72].

Even without the consideration of receptor flexibility, the vast search space resulting from the combination of possible conformations and docking poses typically makes an exhaustive search infeasible without extensive prior filtering. Generic meta-heuristics are therefore often applied to explore possible docking solutions more efficiently, e.g. Monte Carlo approaches (used in RosettaLigand [73], GlamDock [74], GLIDE [34] and LigandFit [75], among others) or Evolutionary Algorithms (used in GOLD [76], FITTED [77], BetaDock [62] and FLIPDock [71]). An alternative search method derived from *de* *novo* ligand design is the Incremental Construction approach [78], which first places a base fragment or anchor fragment of the ligand in the binding pocket and then adds the remaining fragments incrementally to fill cavities, considering different possible solutions resulting from conformational flexibility (e.g. used in FlexX [66, 78], Dock [60,61] and Surflex [79]). More recently, docking approaches using an exhaustive search within multi-step filtering approaches for docking poses have been proposed, e.g. using reduced-resolution shape representations and a smooth shape-based scoring function (FRED [80]), or applying a new graph matching algorithm to enumerate all compatible pose combinations of rigid sub-fragments from a decomposed ligand (eHITS [81, 82]). Table 3 provides an overview of currently available free and commercial protein–ligand docking programs and the main algorithmic principle used, highlighting that a wide selection of current approaches is already freely available for academic research.

**Table 3.** Software tools for protein-ligand docking with information on the main algorithmic principle used and the public accessibility

Software	Principle	Free for academia	Webpage
AutoDock [63, 64]	Monte Carlo & Lamarckian genetic algorithm	Yes	http://autodock.scripps.edu/
AutoDock Vina [130]	Iterated local search	Yes	http://vina.scripps.edu/
DOCK [60, 61]	Incremental construction	Yes	http://dock.compbio.ucsf.edu/
SLIDE [131, 132]	Mean field theory optimization	Yes	http://www.bch.msu.edu/∼kuhn/software/slide/index.html
RosettaLigand [73]	Monte Carlo	Yes	http://rosettadock.graylab.jhu.edu/
FRED [80]	Exhaustive search multi-step filtering	Yes	http://www.eyesopen.com/oedocking
FITTED [77]	Genetic algorithm	Yes (no cluster use)	http://www.fitted.ca
GlamDock [74]	Monte Carlo	Yes	http://www.chil2.de/Glamdock.html
SwissDock / EADock DSS [133, 134]	Exhaustive ranking & clustering of tentative binding modes	Yes	http://www.swissdock.ch/
iGEMDOCK / GEMDOCK [135]	Evolutionary algorithm	Yes	http://gemdock.life.nctu.edu.tw/dock/igemdock.php
rDOCK [136]	Genetic algorithm + Monte Carlo + simplex	Yes	http://rdock.sourceforge.net/
BetaDock [62]	Genetic algorithm	Yes	http://voronoi.hanyang.ac.kr/software.htm
FLIPDock [71]	Genetic algorithm	Yes	http://flipdock.scripps.edu/
GalaxyDock2 [137]	Conformational space annealing	Yes	http://galaxy.seoklab.org/softwares/galaxydock.html
LeadIT (FlexX/HYDE) [66, 114]	Incremental construction	No	http://www.biosolveit.de/leadit/
GLIDE [34]	Side point search + Monte Carlo	No	http://www.schrodinger.com/
GOLD [76]	Genetic algorithm	No	http://www.ccdc.cam.ac.uk/Solutions/GoldSuite/Pages/GOLD.aspx
Surflex [79, 107]	Incremental construction	No	http://www.tripos.com/index.php
ICM [138, 139]	Iterated local search	No	http://www.molsoft.com/docking.html
MOE [84]	Parallelized FlexX (see above)	No	http://www.chemcomp.com/MOE-Molecular_Operating_Environment.htm
LigandFit [75]	Monte Carlo	No	http://accelrys.com/products/discovery-studio
eHiTS [81, 82]	Exhaustive search multi-step filtering	No	http://www.simbiosys.ca/ehits/index.html
Drug Discovery Workbench [140, 141]	Multiple metaheuristics	No	http://www.clcbio.com/products/clc-drug-discovery-workbench

The broad range of structure representation and search methodologies covered by these software tools is complemented by an equally wide variety of scoring functions used to evaluate docking poses. These can roughly be grouped into three types of approaches: (1) classical molecular mechanics or force field-based methods (e.g. adjusted force fields like AMBER [93] and CHARMM [86] and variants applied in DOCK [60, 61], GoldScore [76, 98] and AutoDock [63, 64]); (2) empirical scoring functions, obtained via regression analysis of experimental structural and binding affinity data (e.g. ChemScore [99], FlexX/F-Score [102], X-Score [103], GlideScore [34], LUDI [65], PLP [104], Cyscore [105], ID-Score [106] and Surflex [79, 107]; and (3) knowledge-based scoring functions, derived using information from resolved crystal structures (e.g. DrugScore [108], DSX [109], PMF [110], ITScore [94], SMoG [111], STScore [112] and ASP [113]). Moreover, many *in silico* screening pipelines have extended these fast-ranking approaches by applying a refined but more time-consuming scoring as a post-processing to only the top-ranked poses, e.g. using methods for absolute binding affinity estimation [114–116].

To give the user an overview of the typical predictive performance and runtime efficiency to be expected from commonly used receptor-based screening approaches, a variety of comparative reviews have been conducted. Docking performance is typically measured via the enrichment factor, i.e. for a given fraction x% of the screened compound library, this factor corresponds to the ratio of experimentally found active structures among the top x% ranked compounds to the expected number of actives among a random selection of x% compounds. When comparing different docking methods on benchmark data with known actives, the enrichment factors for the top 1%, 5% and 10% of ranked compounds vary significantly across different targets (e.g. depending on the protein family, the quality of the crystal structure and the drugability of its binding pocket) and different docking methods, ranging from between 1.6 to 14.8 with a median enrichment factor of 4 in a large-scale validation study (always using the best-performing scoring function available for each docking method) [117]. However, no method was consistently superior to other approaches across different data sets. A separate comparative study plotted the rate of true-positive identifications against the rate of false-positives for different docking approaches and benchmark data sets to determine the area under the curve (AUC) as a performance measure [92]. Mean AUC values between 0.55 and 0.72 were obtained, and the GLIDE HTVS approach [34] significantly outperformed other methods. Instead of relying on published evaluation studies, users can also evaluate their own docking pipeline on one of the widely used benchmark collections, e.g. the Directory of Useful Decoys [118] and Maximum Unbiased Validation [119]. Apart from the predictive performance, the runtime requirements for docking simulations also vary largely depending on the size and conformational flexibility of ligand(s) and the binding pocket (or the protein surface for blind docking), and the structure representation, scoring and search space exploration approach used. To alleviate the computational burden resulting from a runtime behaviour that tends to scale exponentially with the number of degrees of freedom to be explored, docking algorithms use efficient sampling techniques [102, 120] and search space exploration methods (e.g. divide-and-conquer or branch-and-bound [97, 120]), and prior knowledge to prune the search space, e.g. from rotamer libraries [70, 71]. Moreover, some docking algorithms have been parallelized [121, 122] or extended to exploit GPU acceleration [123, 124] and FPGA-based systems [124, 125]. On a common mono-processor Linux workstation, typical software tools dock up to 10 compounds per second [126, 127], but to obtain reliable runtime estimates, the user should perform test runs on a few representative compounds for the library to be screened. In any case, the user will need to take into consideration that the achievable quality and efficiency of docking algorithms will always be subject to general limitations, resulting from the restricted quality of the input receptor structure(s), the total number of degrees of freedom for fully flexible docking and the inaccuracies of *in silico* scoring functions.

Apart from classical docking approaches, in recent years, several software packages have also complemented conventional screening for non-covalent interactions by dedicated covalent docking methods, e.g. DOCKTITE [83] for the MOE package [84], CovalentDock [85] for AutoDock [63, 64], CovDock [87] for GLIDE [34] and DOCKovalent [88] for DOCK [60, 61]. These approaches typically first identify nucleophilic groups in the target protein and electrophilic groups in the ligand and then apply similar search space exploration methods as in classical docking, using dedicated scoring terms to account for the energy contribution of covalent bonds (however, often the user first has to specify an attachment site, e.g. a cysteine or serine residue in the binding pocket).

A further more recent development is the use of consensus ranking and machine learning techniques to combine either the final outcomes for different docking methods or integrate different components of their scoring functions to obtain a more reliable assessment of docking solutions [89–91, 95]. These integration techniques outperform individual algorithms in the great majority of applications, suggesting that users should ideally not rely on only a single docking approach or scoring function. Using parallel processing on high-performance computing systems, such integrative compound rankings across different methods can be obtained without significantly extending the overall runtime.

Finally, new drug design techniques have been developed to account for protein mutations that may confer drug resistance, e.g. in cancer cells and viral or bacterial proteins. Generally, two types of strategies can be distinguished: (1) approaches directly targeting the mutant proteins with drug resistance; and (2) approaches using single drugs or drug combinations targeting multiple proteins. Combinatorial therapies using multiple drugs with multiple targets have become a standard for the treatment of HIV infections, and statistical learning software to predict optimal drug combinations from the HIV genome sequence, in particular the Geno2Pheno approach [96], is already applied in clinical practice. Directly targeting mutant proteins is a more challenging task, as crystal structures of the mutants are usually not available and difficult to model *in silico*. Hao *et al*. propose an interesting strategy to use conformational flexibility within inhibitor structures to address drug resistance, focusing on the HIV-1 reverse transcriptase target [100]. However, as increased structural flexibility may also result in reduced selectivity, most published approaches to counteract drug resistance use conventional small-molecule design techniques, but exploit detailed knowledge on the structural basis of resistance for specific targets to prioritize ligands in terms of the likelihood of binding robustly to different mutated variants of the target. For example, Esser *et al*. analyse where and why inhibitors for the respiratory component cytochrome bc1 complex subunit fail and propose alternatives by considering different active sites in the protein [101]. For kinase targets in cancer diseases, Bikker *et al*. discuss patterns formed by the location of resistance mutations across multiple targets and their implications for drug design [128]. Apart from mutations, other types of drug resistance mechanisms, e.g. over-expression of efflux transporters in cancers, have previously been reviewed in detail [129].

### Ligand-based screening

A receptor structure of sufficient quality for docking simulations is often not available for a chosen target protein. Alternatively, if binders for the target binding pocket are already known, further compounds may be predicted as binders with similar type of activity from their structural and chemical similarity to the known ligands. In analogy to the previously discussed docking methods, corresponding ligand-based screening techniques differ in terms of structure representation, consideration of structural flexibility and the used search methodology and scoring function.

To represent structures compactly for fast similarity searches, a wide variety of molecular descriptors has been proposed, including *0D-descriptors* (simple count and constitutional descriptors like atom count, bond count and molecular weight); *1D-descriptors* (binary fingerprints for the presence/absence of structural features, fragment counts and rule-based sub-structure representations known as SMILES/SMARTS [142]); *2D-descriptors* (topological descriptors / graph invariants like connectivity indices, as well as feature trees [143], see discussion below); *3D-descriptors* (geometry, surface and volume descriptors like 3D-WHIM [144] and 3D-MORSE [145]); and *4D-descriptors* (stereoelectronic and stereodynamic descriptors, obtained from grid-based quantitative structure activity relationship [QSAR] methods like CoMFA/COMSIA [146, 147] implemented in Open3DQSAR [148], or dynamic QSAR techniques covering time-dependent 3D-properties like conformational flexibility and transport properties [149]). A detailed compendium of molecular descriptors has recently been compiled by Todeschini and Consonni [150].

The scoring method to quantify the structural similarity mainly depends on the used descriptor types and individual choices on how to weigh the relevance of different molecular features. For binary fingerprint descriptors, compound similarity is often quantified using the Tanimoto coefficient, i.e. the proportion of the features shared among two molecules divided by the size of their union (similar scores include the Dice Index and Tversky Index with adjustable weights, see [151] for a comparison of different approaches). More recently, similarity scoring using data compression and the information-theoretic concept of the Normalized Compression Distance [152] has been proposed for string-based molecule representations (implemented in the software Zippity [153]). To account for both topological and physicochemical properties, Rarey and Dixon introduced a fast screening approach using feature trees, a graph-based representation of molecular sub-fragments and their interconnections [143]. While these techniques relying on 1D- and 2D-descriptors are suitable for screening millions of compounds, more complex scoring functions using 3D- and 4D-descriptors, statistical learning and available binding affinities for already known binders can provide more accurate estimations, but involve significantly higher runtimes (i.e. they are mainly suitable for post-screening of pre-selected compounds). In particular, using more computationally expensive algorithms for flexible ligand superposition, compounds can be overlaid onto known binding molecules by matching their shape and functional groups (e.g. implemented in Catalyst/HipHop [154], SLATE [155], DISCO [156], GASP [157], GALAHAD [158], GAPE [159] and PharmaGIST [160]) or by superimposing their fragments incrementally onto a template ligand kept rigid, as in FlexS [161]. The superposition of known binders can also enable the inference of a *pharmacophore*, i.e. the 3D arrangement of functional groups and structural features relevant for the binding interactions with the receptor, providing useful constraints to restrict the screening search space. Moreover, if a sufficiently large and diverse training set of known binders is available, sharing the same binding pocket and binding mode, the superimposition of new compounds may enable the prediction of their most likely binding conformations and affinities via machine learning and 3D-QSAR methods (e.g. COMFA and COMSIA [146, 147]). Overall, the choice of molecular descriptors depends on the envisaged application, the available data and runtime for the analysis. Previous comparative reviews may help users to select adequate descriptors and associated analysis techniques (see [162] for a review on descriptors for fast ligand-based screening, and section Protein structure pre-processing and quality control in [163] for a comparison of descriptor-based methods for binding affinity prediction). As an additional filter for a pre-selection of candidate descriptors, statistical feature selection methods can be applied [164]. The reader should also note the generic limitations of different descriptor types; in particular, 1D- and 2D-descriptors can only capture limited and indirect information on the spatial structure of ligands, whereas the descriptors used in 3D-QSAR methods like COMFA and COMSIA overcome this restriction at the expense of necessitating a computationally complex ligand superpositioning [163]. Descriptors for dynamic 3D properties like conformational flexibility cover an additional layer of information not sufficiently addressed by simpler descriptor types [149]; however, the amount and type of data required to calculate these descriptors limits their applicability. Apart from the type of information captured by descriptors, their interpretability may also be considered as a selection criterion (e.g. topological indices [165] have been criticized for a lack of a clear physicochemical meaning). As the number of proposed descriptors continues to grow and no simple rules are available to choose optimal descriptors for each application, users may also wish to consult dedicated reference works explaining and comparing descriptor properties in detail [150].

Moreover, performance evaluations have been conducted on benchmark data to compare ligand-based screening methods using different descriptors against receptor-based screening techniques. Interestingly, in many of these studies, ligand-based methods have been reported to provide either similar or better enrichment of actives among the top-ranked compounds [117, 126, 166]. For example, Venkatraman *et al*. found that 2D fingerprint-based approaches provide higher enrichment scores than docking methods for many targets in benchmark data sets [166]. However, as most ligand-based screening approaches score new compounds in terms of their similarity to already existing binders, the novelty of top-ranked molecules may often be limited as compared with new binders identified via docking approaches. From their results, Venkatraman *et al*. also derive the recommendation to use descriptors that can represent multiple possible conformations of a ligand. Another comparative study by Krüger *et al*. obtained comparable enrichments with approaches based on receptors or ligands, but diverse performance results were observed across different groups of targets [117]. Therefore, the authors suggest to consider both types of approaches as complementary and, if possible, apply them jointly to increase the number and structural variety of identified actives. Indeed, a comparison of data fusion techniques to combine screening based on receptors or ligands by Sastry *et al*. [92] showed that the average enrichment in the top 1% of ranked compounds could be improved by between 9 and 25% in comparison to the top individual approach for different benchmark data sets (with a mean enrichment factor between 20 to 40).

One of the main advantages of ligand-based screening methods using 0D-, 1D- and 2D-descriptors are their extremely short runtimes, e.g. fingerprint similarity searches can screen around 10 000 ligands per second on a 2.4-GHz AMD Opteron processor [92]. For comparison, on the same processor, 3D-ligand based methods like shape screening can screen roughly 10 ligands per second on a database of pre-computed conformations, and docking with Glide HTVS takes approx. 1–2 s per ligand [92]. However, the applicability of ligand-based screening methods is strictly limited by the availability, number and diversity of known binding ligands for the target and specific binding pocket of interest, and the most widely used similarity-based scoring functions will by design only find compounds with high similarity to already known binders.

In summary, although most ligand-based approaches are not designed to identify entirely new binders with diverse structures and binding modes, structurally similar compounds to known binders may still display improved properties in terms of affinity, selectivity or ADMETox properties, as exemplified by previous success stories [167–169]. Finally, if both the receptor structure and an initial set of known binders are available for the target protein, the combination of screening techniques based on receptors or ligands may help to increase the enrichment of active molecules among the top-ranked compounds [170].

### ADMETox and off-target effects prediction

In preclinical drug development projects, screening using docking or ligand similarity scoring is often applied in combination with *in silico* methods to estimate bioavailability, selectivity, toxicity and general pharmacokinetics properties to filter compounds more rigorously before final experimental testing. While simple rules to evaluate ‘drug-likeness’ and oral bioavailability like ‘Lipinski’s rule of five’ and similar rule sets [57, 58] already enable a fast pre-selection of compounds, machine learning techniques provide opportunities for more accurate and detailed assessments of a wider range of outcome measures. The computational prediction of ADMETox properties (i.e **A**bsorption, **D**istribution, **M**etabolism, **E**limination and **Tox**icity properties) is therefore gaining increasing attention.

For this purpose, quantitative structure-property relationship (QSPR) models, i.e. regression or classification models relating molecular descriptors to a target property of interest, have been developed to predict various pharmacokinetic and biopharmaceutical properties. While classical QSPRs are mostly designed as simple linear models depending on only a few descriptors, more recently, advanced statistical learning methods combining feature selection with support vector machines, partial least squares discriminant analysis and artificial neural networks have been used to build more reliable ADMETox prediction models [171, 172]. To evaluate and compare different models, performance statistics like the mean cross-validated accuracy or squared error, the standard deviation and Fisher’s *F*-value can be used (see [173] for a review of QSPR validation methods).

Apart from QSPR models, rule-based expert systems like METEOR [174], MetabolExpert [175] and META [176] use large knowledge bases of biotransformation reactions to provide rough indications of the possible metabolic routes for a compound. Expert systems have also been proposed to combine large collections of rules for toxicity prediction, as QSPR models are mostly limited to specific toxicity endpoints. Changes in a single reactive group can turn a non-toxic into a toxic compound and long-term toxicities are generally difficult to identify and study; hence, the available prediction software mainly focuses on established fragment-based rules for acute toxicity (relevant software includes COMPACT [177], OncoLogic [178], CASE [179, 180], MultiCASE [181], Derek Nexus [182], TOPKAT [183], HazardExpert Pro [184], ProTox [185] and the open-source Toxtree [186]).

A further option to identify adverse effects resulting from off-target binding are inverse screening approaches, screening a ligand against many possible receptor proteins using docking or similarity scoring to their known binders. Fast heuristic approaches for this purpose include idTarget [187], TarFisDock [188], INVDOCK [189], ReverseScreen3D [190], PharmMapper [191], SEA [192], SwissTargetPrediction [193] and SuperPred [194].

Overall, current *in silico* ADMETox modelling and prediction methods are still limited in their accuracy and coverage for estimating biomedically relevant compound properties, but may provide useful preliminary filters to exclude subsets of compounds with high likelihood of being toxic or having insufficient bioavailability.

## Generic screening framework and workflow management

**Figure 1.**
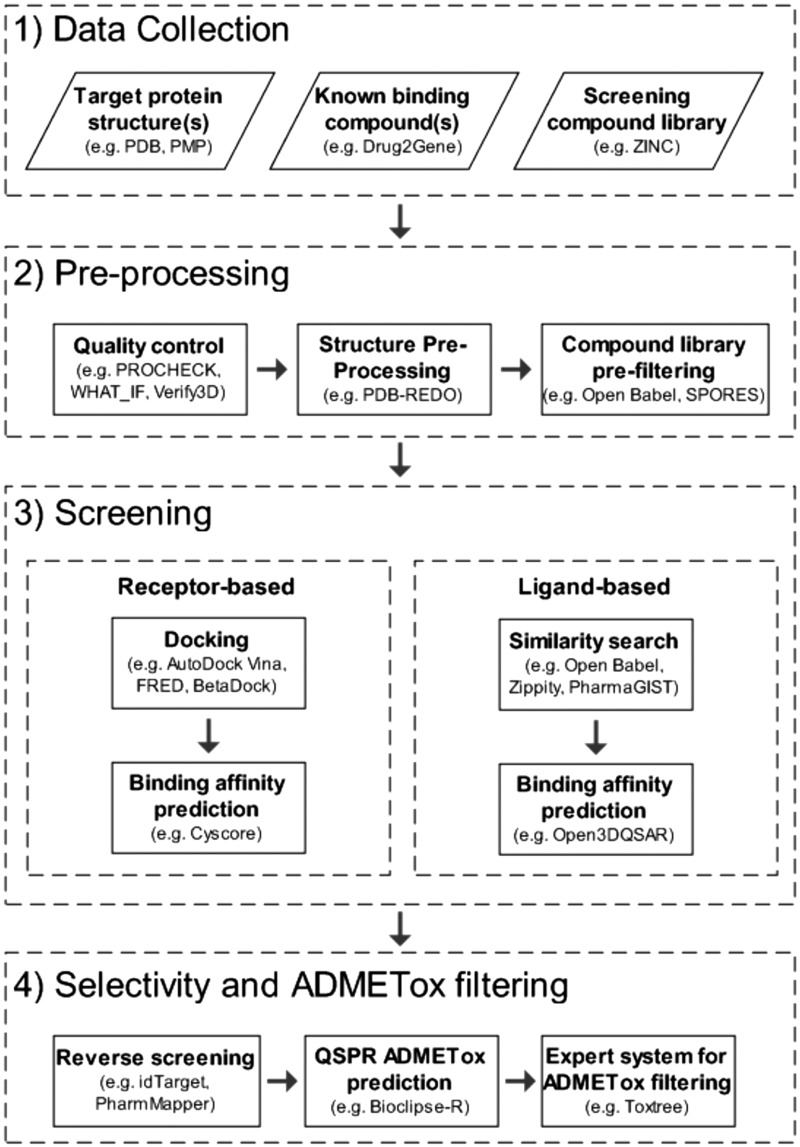
Generic framework for *in silico* small-molecule screening (examples of free software tools for each step are listed in brackets).

Implementing an *in silico* screening project for preclinical drug development requires the set-up of a complex analysis pipeline, interlinking multiple task-specific software tools in an efficient manner. In Figure 1, a generic screening framework is shown, covering the typical steps in computational small-molecule screening projects and providing examples of free software tools for each task.

The four main phases in the framework, (1) data collection, (2) pre-processing, (3) screening and (4) selectivity and ADMETox filtering, are common across different projects and sub-divided into more specific sub-tasks for data collection and pre-processing, whose implementation will depend on the available resources, the chosen strategy and study type (e.g. differing for screening studies based on receptors or ligands).

To implement a corresponding software pipeline and facilitate the interlinked, reproducible and automated application of screening software, various workflow management tools have been developed over the past years. The most widely used systems in structural bioinformatics are the open-source software KNIME [195, 196] and the commercial Pipeline pilot (Accelrys), but several other tools exist, including Taverna [197], KDE Bioscience [198], Galaxy [199], Kepler [200], VisTrails [201], Vision [202], Triana [203] and SOMA2 [204]. These approaches mainly differ in terms of the supported level of parallelism, e.g. in KNIME, a new task can only start after completing the preceding one, whereas in pipelining tools like Pipeline pilot, task operations continue on the next records in the data stream while already processed data records are passed on to the next task. Pipelining approaches often have advantages in terms of efficiency; however, the workflow methodology used in KNIME may make it easier for the user to inspect intermediate outputs, identify task-specific issues and resume the execution of interrupted workflows (e.g. after a power-cut).

KNIME also supports the integration of different databases (e.g. MySQL, SQLite, Oracle, IBM DB2, Postgres) to load, manipulate and store data efficiently, and similarly, Pipeline pilot can integrate standard databases via the Open Database Connectivity (ODBC) protocol (specifically, for the integration of molecular and biological databases, templates are already available). Due to the small sizes of ligand files and the limited space required to store compressed numerical screening data, the total disk space required for a screening study is typically not a major limiting factor with current hard disk capacities; in particular, because workflow management tools like KNIME are able to store only the differences between consecutive nodes. However, frequent disk-access operations can slow down the execution of screening workflows. The available options to address this issue include data caching, in-memory storage and the use of efficient database queries. Thus, workflow management systems like KNIME and Pipeline pilot are not meant to replace database systems for effective storage and retrieval of screening results, but rather integrate these databases and provide additional features to simplify the set-up, monitoring, adjustment and sharing of screening workflows.

Other workflow management systems are mostly used for different applications, but partly also provide dedicated features for virtual screening. For example, the free Taverna system can be interlinked with the open-source cheminformatics Java library CDK [205] and the Bioclipse workbench [206] for QSAR analyses and molecular visualizations. Some of the systems are designed specifically for visual data exploration and users with limited programming experience, allowing the set-up of complex workflows and subsequent data analysis in an almost purely visual manner, e.g. Vision [202] and VisTrails [201]. Together with Taverna, VisTrails also stands out for its strong focus on data reproducibility and provenance management.

The set-up of reproducible screening pipelines can also be facilitated via open virtualization platforms to run distributed applications, e.g. the Docker platform (https://www.docker.com). As a complementary software to this review article, a downloadable cross-platform system for reproducible virtual screening using Docker has been implemented and made publicly available for the reader (https://registry.hub.docker.com/u/vscreening/screening). It integrates several free tools covering the different phases of the proposed generic framework for screening based on receptors or ligands, e.g. OpenBabel [52] for file format conversions and filtering, AutoDock Vina [63, 64] for molecular docking, CyScore [105] for binding affinity prediction and ToxTree [186] to estimate toxicity hazards, among various others (see https://registry.hub.docker.com/u/vscreening/screening for details). A script to run an example screening for inhibitors of HIV-1 protease using compounds from the NCI Diversity Set 2 [207] is also provided, and the user can simply change the input files to study alternative targets and compound libraries.

In summary, workflow management and virtualization tools provide new means to obtain reproducible and portable screening pipelines, which can be adjusted and extended with minimal effort. The framework and software proposed here may serve as a starting point to test and compare combinations of different public tools, or to expand and alter the framework to meet the goals of a specific new screening project.

## Conclusion

Virtual small-molecule screening is still a highly challenging task with many possible pitfalls, e.g. due to errors in the input structures and limitations in the scoring and search space exploration methods. However, as highlighted in the generic framework for *in silico* screening presented here, free software and relevant public databases have now become available for each common task in a screening project. This is partly due to the recent expiration of patent protection for some fundamental cheminformatics techniques (e.g. CoMFA [146]), but mainly due to a growing open-source community, developing frequently updated and freely modifiable screening tools. More recently, such non-proprietary software alternatives are also becoming more widespread for the workflow management of complex screening pipelines on diverse computing platforms. As a result, efficient and reproducible screening workflows can now be implemented at lower cost and effort, making preclinical drug research projects more feasible within an academic setting.

Key Points

- A wide range of free tools and resources for each common task in virtual small-molecule screening have become available in recent years. These tools can be combined into professional screening pipelines using typical hardware facilities in an academic environment.
- Molecular structure files from public databases are usually not pre-processed for virtual screening purposes. In particular, PDB files for protein crystal structures are often affected by several errors and missing residues. Therefore, care must be taken to apply adequate pre-processing and quality control methods during the initial stages of a screening project.
- Workflow management systems can greatly facilitate the set-up, monitoring and adjustment of virtual screening pipelines. They allow users to build reproducible workflows that can be scaled from desktop systems to high-performance, grid and cloud computing platforms.

## Funding

This work was supported by the Fonds Nationale de la Recherche, Luxembourg (grant no.: C13/BM/5782168).
